# The Long-Term (3.5-Year) Observation of Asymptomatic Sclerosing Mesenteritis: A Case Report

**DOI:** 10.7759/cureus.69960

**Published:** 2024-09-22

**Authors:** Hiroki Takiyama, Tsutomu Nishida, Dai Nakamatsu, Kengo Matsumoto, Masashi Yamamoto

**Affiliations:** 1 Department of Gastroenterology, Toyonaka Municipal Hospital, Toyonaka, JPN

**Keywords:** asymptomatic, benign course, long-term observation, mesenteric mass, sclerosing mesenteritis

## Abstract

A 57-year-old male with a history of hypertension, diabetes mellitus, and dyslipidemia was found to have elevated carcinoembryonic antigen (CEA) levels during a routine health checkup, leading to an abdominal computed tomography (CT) scan. The scan identified a mesenteric mass with an irregular morphology. Subsequent blood tests indicated no signs of inflammation, and follow-up CEA levels normalized. Further imaging with abdominal contrast-enhanced CT and 18F-fluorodeoxyglucose positron emission tomography (FDG-PET)/CT revealed a calcified mass in the mesentery, raising concerns for malignancy. However, an exploratory laparotomy and biopsy confirmed the diagnosis of sclerosing mesenteritis (SM). During a 3.5-year period, the patient remained asymptomatic, with serial imaging showing no significant changes in the mass, even without treatment. This case underscores the potential benign course of SM, suggesting that conservative management may be appropriate in select asymptomatic cases.

## Introduction

Sclerosing mesenteritis (SM) is a rare, benign inflammatory condition primarily affecting the mesentery of the small intestine, characterized by fat necrosis, chronic inflammation, and fibrosis [1,2]. Although its etiology remains unclear, SM is hypothesized to be secondary to factors such as abdominal surgery, trauma, autoimmune processes, infection, ischemia, or malignancy [2]. It is also proposed that SM occurs in genetically predisposed individuals, where trauma triggers abnormal connective tissue healing and repair responses [3]. Most cases of SM are discovered incidentally during imaging studies, as patients are often asymptomatic. However, in some cases, the compressive effects on the intestines, lymphatic vessels, and blood vessels can lead to symptoms such as bowel obstruction, chylous ascites, or mesenteric ischemia [4]. These can result in a variety of gastrointestinal and systemic symptoms, including abdominal pain, nausea and vomiting, diarrhea, weight loss, and fever [3]. Despite numerous case reports [5] and series [1], the natural history of asymptomatic SM remains poorly understood, with limited long-term observational data. This case report describes a rare instance of asymptomatic SM observed over 3.5 years without progression and without treatment, offering valuable insight into the potential benign nature of the disease in such cases.

## Case presentation

**Figure 1:**
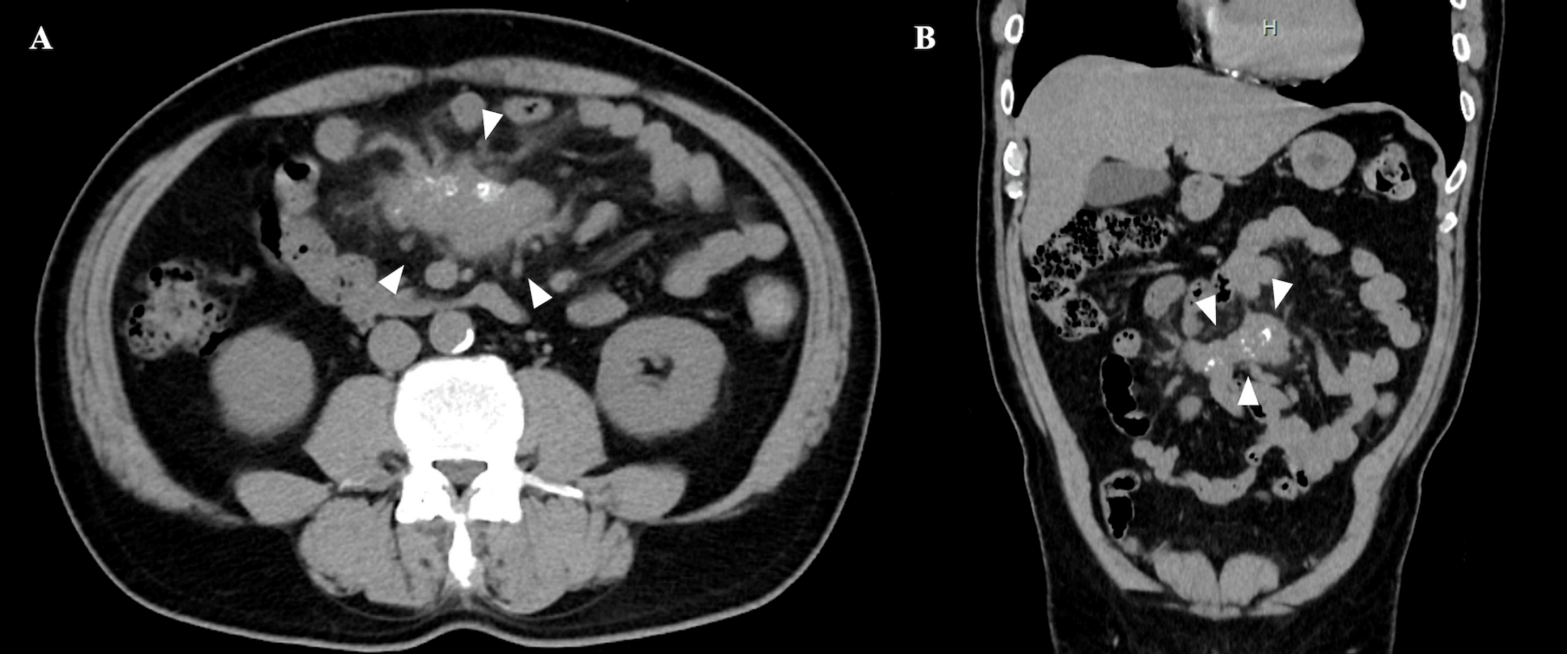
Plain abdominal computed tomography. An irregular mass, measuring 5.5 cm in length with calcification, is seen in the mesentery of the small intestine (A, horizontal section; B, coronal section). The mass is indicated by white triangles. The mesentery surrounding the lesion also shows ciliary shadows, with extensive contact with the small intestine.

A 57-year-old male with a history of hypertension, diabetes mellitus, and dyslipidemia was found to have elevated carcinoembryonic antigen (CEA) levels during routine follow-up at a nearby clinic. His CEA level had increased from 2.9 to 7.2 ng/mL, prompting an abdominal plain computed tomography (CT) scan that detected an irregular mass measuring 5.5 cm in the mesentery of the small intestine (Figure 1).

The patient had no relevant history or related comorbidities. The patient was referred to our department in November 2020 for further evaluation. His medical history was notable for congenital optic nerve atrophy (amblyopia) and glaucoma, which led to complete blindness around the age of 47. His family history included liver cancer in his father (specific details unknown) and pleural cancer in his mother. The patient was on multiple medications, including ethyl icosapentate, loratadine, omeprazole, metformin/sitagliptin, nifedipine, atenolol, azilsartan, and pravastatin. Regarding social history, the patient consumed alcohol but did not smoke. He had no known drug allergies.

At the initial consultation, the patient did not report any noticeable symptoms such as abdominal pain. Blood tests revealed a white blood cell count of 8,600/μL and a C-reactive protein (CRP) level of 0.09 mg/dL, indicating no signs of inflammation. Fasting blood glucose levels were elevated, and hemoglobin A1c (HbA1c) level was 6.9%, consistent with known glucose intolerance. No other abnormalities were observed in the biochemical tests. Upon re-examination, the CEA level had normalized to 4.4 ng/mL, and other tumor markers (carbohydrate antigen {CA} 19-9, neuron-specific enolase, and soluble interleukin-2 receptor) were within normal limits (Table 1).

**Table 1:** Laboratory data on admission.

Parameters	At the first visit	Reference range
White cell count (/μL)	8,600	3,300-8,600
Neutrophils (%)	63.8	40-68
Lymphocytes (%)	28.4	26.0-46.6
Red blood cells (×10^4^/μL)	519	389-492
Hemoglobin (g/dL)	15.9	11.6-14.8
Hematocrit (%)	46.8	35.1-44.4
Platelet count (×10^4^/μL)	23.4	15.8-34.8
Prothrombin time (PT) (%)	101	70-130
PT-international normalized ratio	1.00	0.9-1.1
Total protein (g/dL)	7.6	6.6-8.1
Albumin (g/dL)	4.4	4.1-5.1
Aspartate transaminase (U/L)	19	13-30
Alanine transaminase (U/L)	22	7-23
Lactate dehydrogenase (U/L)	151	135-214
Alkaline phosphatase (U/L)	170	98-328
γ-Glutamyl transpeptidase (U/L)	49	9-32
Amylase (U/L)	121	44-132
Blood urea nitrogen (mg/dL)	16	8-20
Creatinine (mg/dL)	0.90	0.46-0.79
Fasting glucose (mg/dL)	143	73-109
Hemoglobin A1c (%)	6.9	4.6-6.2
Sodium (mEq/L)	139	138-145
Calcium (mg/dL)	9.7	8.8-10.1
Total bilirubin (mg/dL)	0.56	0.2-1.2
C-reactive protein (mg/dL)	0.09	<0.3
Carcinoembryonic antigen (ng/mL)	4.4	<5
Carbohydrate antigen 19-9 (U/mL)	16	<37
Soluble interleukin-2 receptor (U/mL)	336.8	156.6-474
Alpha fetoprotein (ng/mL)	2.0	<10
Neuron-specific enolase (ng/mL)	10.4	<16.3

**Figure 2:**
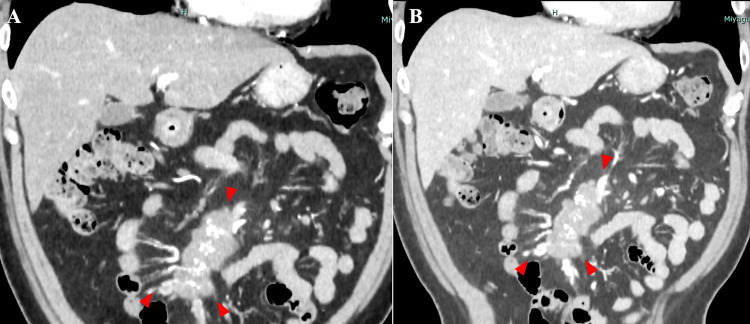
Coronal section of abdominal contrast-enhanced CT scan. The mass, indicated by red triangles, is depicted as hypovascular with poor contrast enhancement. No contrast effect was observed in either the arterial phase (A) or portal venous phase (B). CT: computed tomography

Abdominal contrast-enhanced CT revealed an irregularly shaped mass with calcifications in the mesentery of the small intestine, encasing the mesenteric arteries and veins (Figure 2).

**Figure 3:**
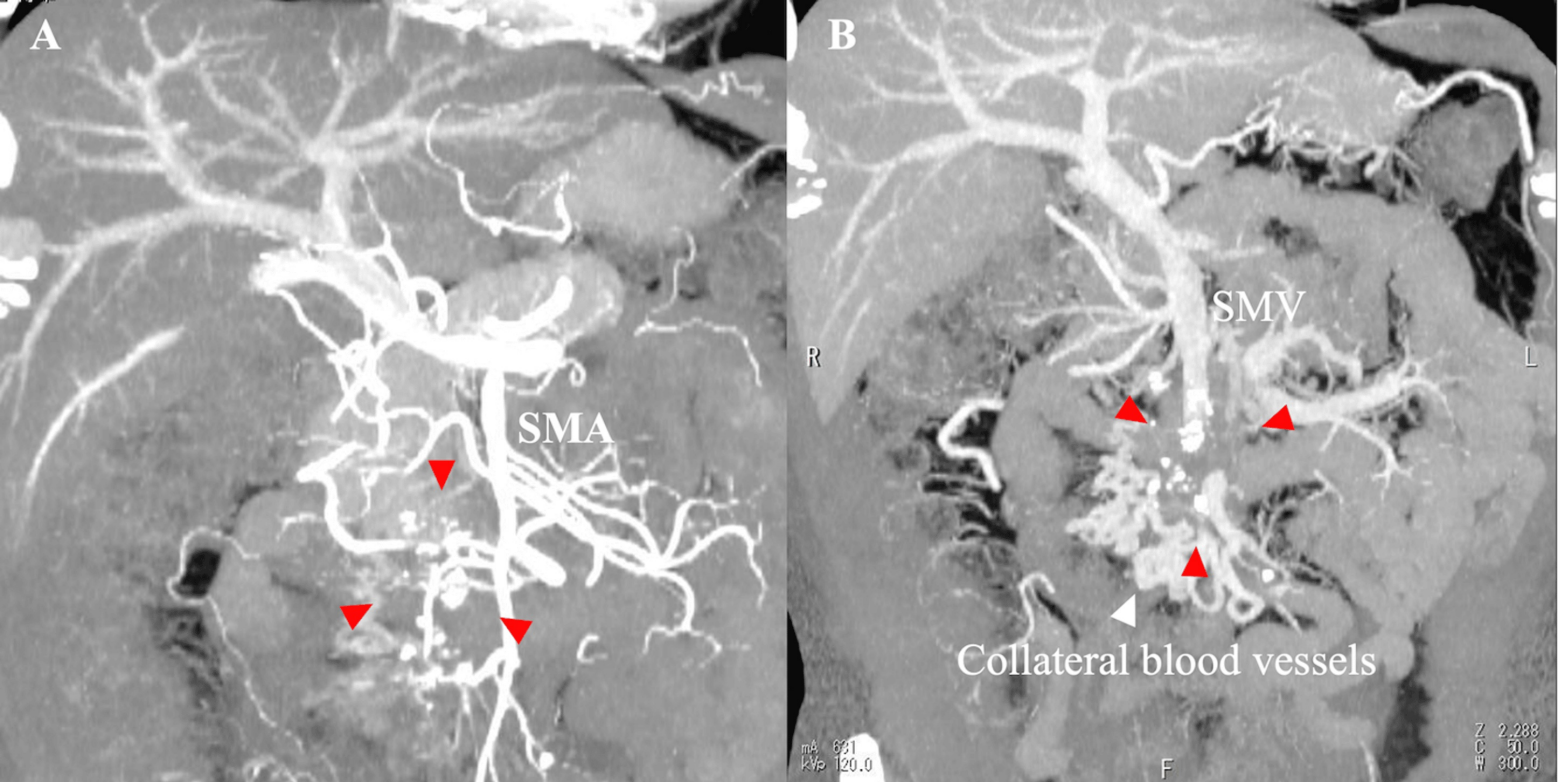
Computed tomography angiography (CTA). CTA revealed a mass, indicated by red triangles, in the arterial phase (A), invading the second and third branches of the superior mesenteric artery (SMA). The superior mesenteric vein (SMV) was occluded in the venous phase, with associated collateral vessels and dilated veins in the surrounding area, indicated by a white triangle.

Computed tomography angiography (CTA) showed a red triangular mass invading the second and third branches of the superior mesenteric artery (SMA) in the arterial phase, with the superior mesenteric vein (SMV) occluded in the venous phase, accompanied by collateral vessels and dilated veins nearby (Figure 3).

**Figure 4:**
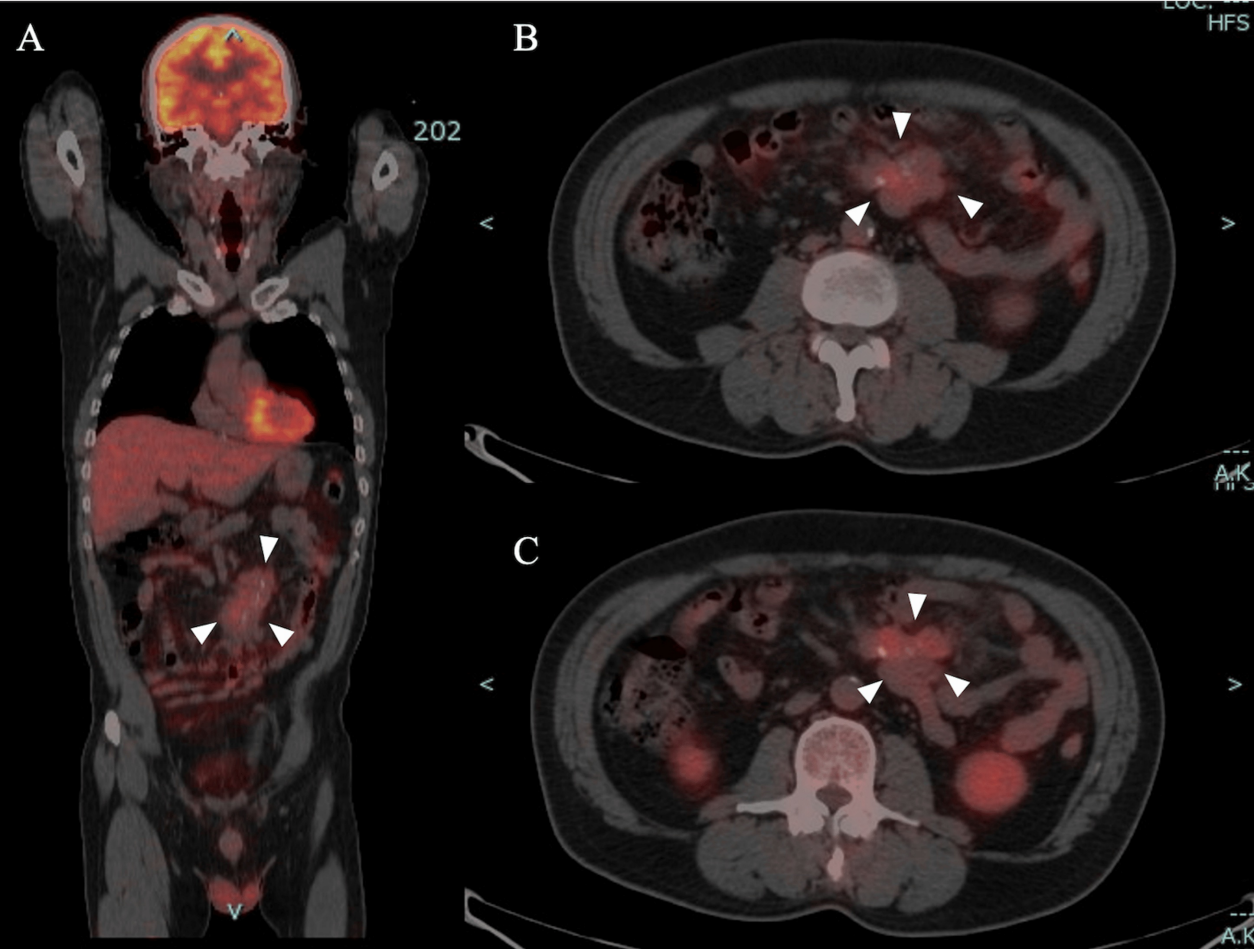
18F-Fluorodeoxyglucose positron emission tomography (FDG-PET)/CT. FDG-PET/CT identified FDG uptake in the mass (A, coronal section; B, horizontal section in the early phase), with a maximum standardized uptake value (SUV) of 3.11 in the late phase (C: delayed phase). CT: computed tomography

18F-Fluorodeoxyglucose positron emission tomography (FDG-PET)/CT revealed FDG uptake in the mass with a maximum standardized uptake value (SUV) of 3.11 in the late phase (Figure 4).

Based on these findings, the differential diagnoses included neuroendocrine tumor, desmoid tumor, mesenteric panniculitis, and SM. Given the encasement of the mesenteric vessels and FDG uptake, malignancy was suspected, prompting an exploratory laparotomy for diagnostic and therapeutic purposes.

**Figure 5:**
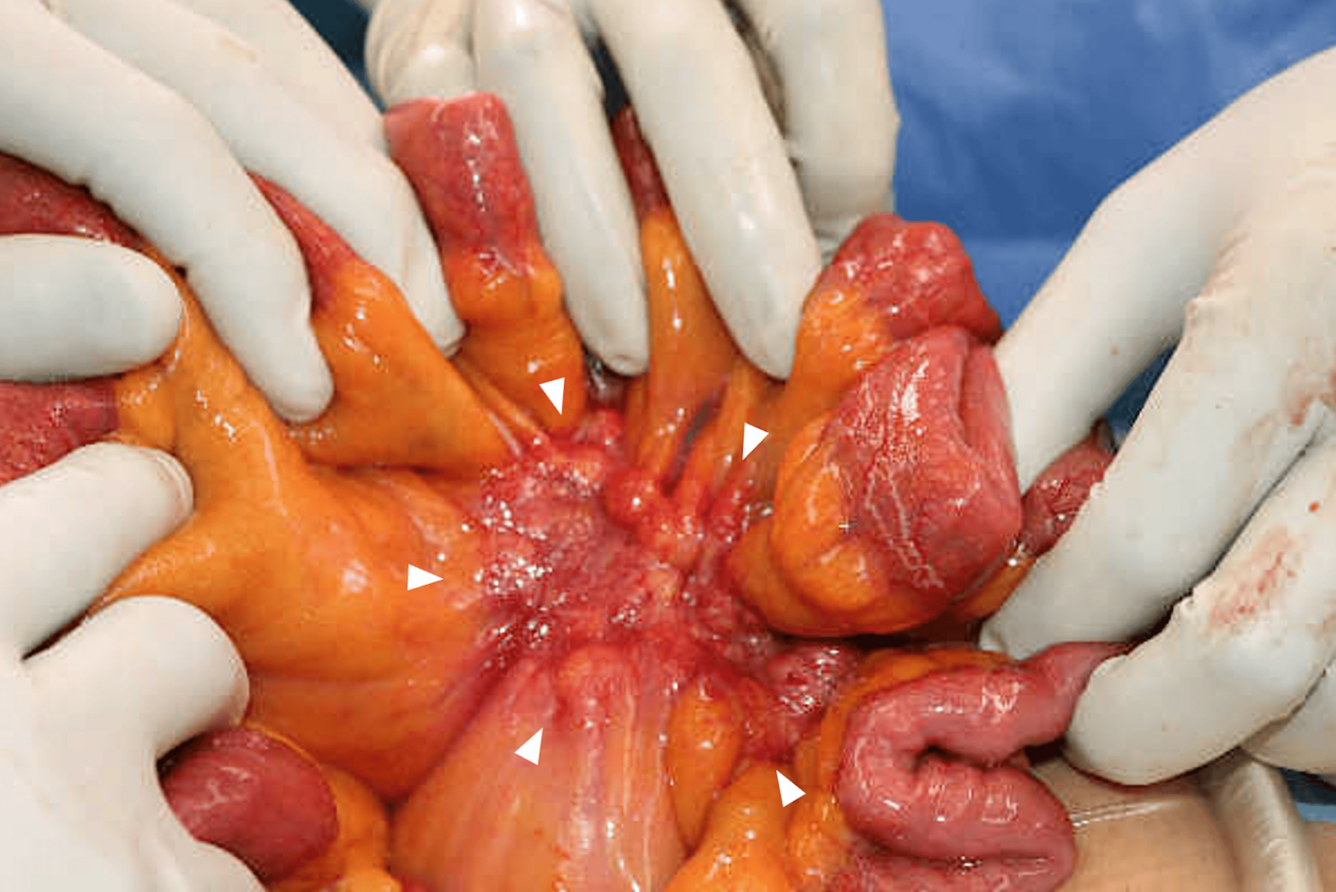
Intraoperative findings. A firm, elastic, ovoid mass with small nodules, suspected to be due to peritoneal dissemination, was observed in the vicinity. The tumors (white triangles) invaded the superior mesenchymal artery and the vein, rendering the mass unresectable. Therefore, a partial resection of the small intestine, including a small portion of the tumor margins, was performed for diagnostic purposes.

Intraoperative findings revealed a firm, elastic, ovoid mass with small nodules, suspected to be peritoneal dissemination, in the vicinity (Figure 5).

**Figure 6:**
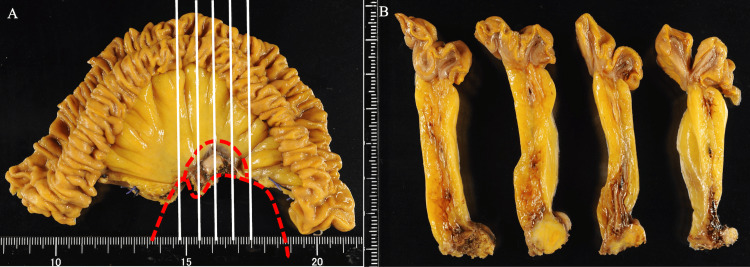
Resected specimen. (A) The mass is indicated by a red dotted line. The area enclosed by the red dotted line contained a small portion of the margins of the mass and was resected. The lesion was excised along a straight line. (B) Cross-sectional view of excised specimen.

Due to the infiltration of the mass into the inferior mesenteric artery and vein, the resection of the mass was deemed unfeasible. The rapid cytological examination of the small nodules suspected of dissemination showed no definitive malignant findings. Therefore, a partial resection of the small intestine, including the tumor margins, was performed for diagnostic purposes (Figure 6).

**Figure 7:**
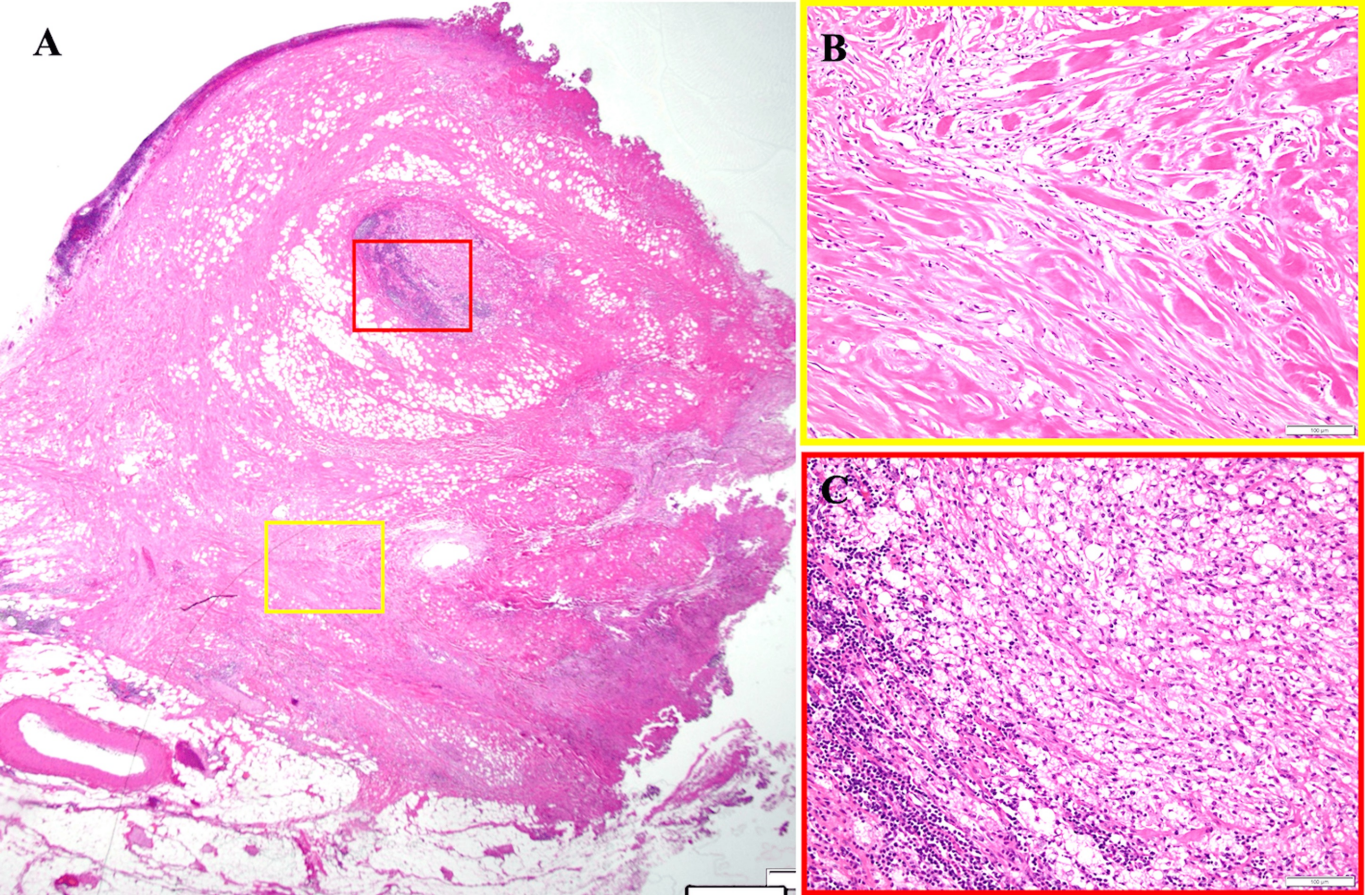
Histopathological findings. Enlarged images of the areas indicated by the yellow and red frames in the photograph in A are shown in B and C, respectively. The findings revealed abundant collagen fibers with hyalinization, the infiltration of inflammatory cells (A), fat necrosis, and macrophages that had phagocytosed fat (B), confirming the definitive diagnosis of sclerosing mesenteritis.

The pathohistological examination of the resected specimen revealed abundant collagen fibers with hyalinization, the infiltration of inflammatory cells, fat necrosis, and macrophages that had phagocytosed fat, leading to a definitive diagnosis of sclerosing mesenteritis (Figure 7).

**Figure 8:**
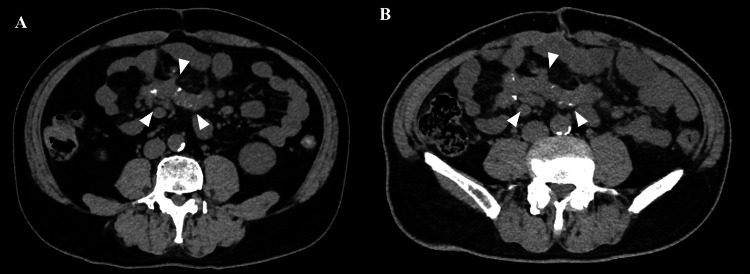
Plain abdominal computed tomography (CT). Plain abdominal CT scans performed at 2.5 years (A: June 2023) and 3.5 years (B: June 2024) after the definitive diagnosis revealed no change in the morphology or size of the branching lobe-shaped lesions (white triangles).

Postoperatively, immunoglobin G (IgG), IgG4, and antinuclear antibody levels were measured to rule out IgG4-related disease, with results of 1,442 mg/dL, 60.4 mg/dL, and less than 1:40, respectively. Since the patient remained asymptomatic, he was kept under observation without treatment. A follow-up CT scan at 2.5 and three years after the diagnosis showed no significant changes (Figure 8).

## Discussion

SM was first identified in 1924 by Jura, who described it as "retractile mesenteritis and mesenteric sclerosis" based on 34 cases [6]. Over the years, SM has been referred to by various names reflecting its histopathological features, such as mesenteric lipodystrophy [7], mesenteric panniculitis [8], and retractile mesenteritis or mesenteric fibrosis [9]. The disease presents with a range of pathological findings, including inflammation, fibrosis, and fat necrosis. These features likely represent different stages in the natural history of the condition, with one feature typically predominating at a particular stage. In 1997, Emory et al. evaluated 84 cases and concluded that these histopathological variations represented a spectrum of the same disease, justifying the use of the term SM [2].

The natural history of SM remains poorly understood owing to the rarity of the disease, inconsistent follow-up, and the various names it has been given over time. SM can manifest with or without symptoms [4,10]. Approximately 20% of the patients with SM develop complications, while the remaining 80% are expected to have a benign, stable, or slowly progressive course [2,11]. However, there are several cases of complications, such as intestinal obstruction [12]. Sharma et al. reviewed 192 patients and found that 1.6% (3/192) of the patients were incidentally diagnosed with SM during imaging studies or surgeries performed for unrelated reasons, suggesting that some cases of SM may remain asymptomatic [11]. Additionally, Daskalogiannaki et al. reported that out of 7,620 consecutive abdominal CT scans performed between January 1995 and March 1998, 49 (0.6%) exhibited findings consistent with SM [13].

The primary goal of SM treatment is symptom relief, with no strong evidence suggesting that treatment significantly improves prognosis, prevents disease progression, or reduces the development of complications. It is estimated that only 1.1%-6.1% of patients identified through imaging require treatment, and most asymptomatic patients remain asymptomatic during follow-up, not requiring intervention [3,13,14]. Therefore, correct diagnosis and appropriate follow-up are crucial, especially when SM is detected asymptomatically, as in our report. However, reports of asymptomatic patients with long-term follow-up are limited. In a study by Akram et al. involving 92 patients, 10% (9/92) were asymptomatic. Of the 44 patients (48%) who received treatment, 26% of the 48 untreated SM patients remained asymptomatic during a median follow-up of 21 months [4].

Lee et al. described a case of SM in a 54-year-old male with hypertension who presented with a large palpable abdominal mass. CT revealed an 18 cm mass in the mesentery of the small intestine [15]. Despite mild abdominal discomfort and no other symptoms, a conservative follow-up was performed. Three months later, a CT scan showed that the mass had shrunk to 9 cm, and the abdominal discomfort had resolved, suggesting spontaneous regression within a few months. Similarly, Hasbahçeci et al. reported a 31-year-old male patient who experienced complete spontaneous resolution of SM within one month without medical or surgical intervention [16]. These cases indicate that spontaneous regression, whether partial or complete, can occur in the SM within a relatively short period. These findings highlight the uncertainty surrounding the prevalence of asymptomatic SM and the long-term outcomes for these patients. In our case, the lesion was discovered incidentally and remained asymptomatic over a 3.5-year observation period without treatment, with no progression or regression on imaging. This suggests that spontaneous regression of SM may occur over a variable period, ranging from approximately one month to more than 10 years, with some cases eventually resolving [17].

The benign nature of SM, particularly in asymptomatic cases, raises questions regarding the necessity and timing of interventions. Some cases of SM may undergo spontaneous regression, as documented in the literature, while others, such as the present case, remain stable over extended periods without intervention. This variability highlights the need for further research to better understand the long-term outcomes of patients with asymptomatic SM and establish evidence-based guidelines for managing this condition.

## Conclusions

This case report highlights a rare instance of asymptomatic SM that was diagnosed incidentally, with no known disease etiology despite thorough interview and examination, and observed over a 3.5-year period without progression or symptom development. In several reported cohort studies, no cases showed an increase in lesion size or worsened during follow-up. These findings suggest that in certain cases, SM may follow a benign course, justifying a conservative management approach. However, further studies are needed to enhance our understanding of the natural history of asymptomatic SM and to provide a more informed basis for clinical decision-making.
